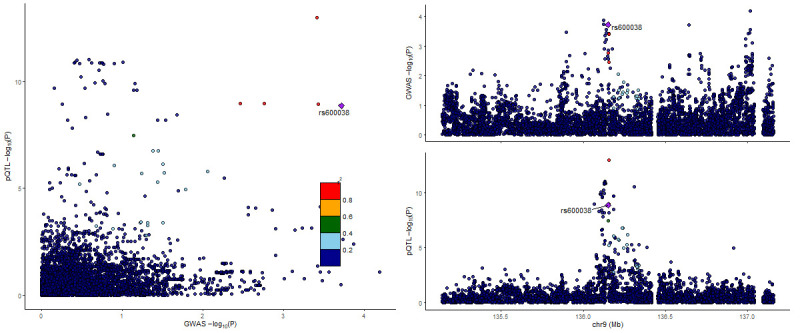# Causal effects of mental disorders on stroke subtypes: A proteome-wide Mendelian randomization and mediation analysis

**DOI:** 10.1097/MD.0000000000049108

**Published:** 2026-06-05

**Authors:** Linhao Cong, Hang Ji, Hailan Yang, Guicheng Kuang, Haitao Liu, Haogeng Sun, Yi Liu

**Affiliations:** a Department of Neurosurgery, West China Hospital, Sichuan University, No. 37 Guoxue Lane, Chengdu, Sichuan, China.

**Keywords:** attention-deficit/hyperactivity disorder, mendelian randomization, mental disorders, metallopeptidase-12, stroke

## Abstract

Observational studies have demonstrated associations between mental disorders and stroke, yet causal relationships and potential molecular mechanisms remain elusive. To clarify this, we performed a bidirectional 2-sample Mendelian randomization (MR) analysis using genome-wide association studies summary statistics to assess the causal effects of 7 major mental disorders (schizophrenia, bipolar disorder, major depressive disorder, autism spectrum disorder, attention-deficit/hyperactivity disorder [ADHD], Alzheimer disease, and Parkinson disease) on stroke subtypes. Analyses were divided into discovery and validation cohorts of European ancestry, followed by MRlap and meta-analysis to obtain pooled estimates. To explore potential circulating protein mediators, 4907 plasma cis-protein quantitative trait loci (cis-pQTLs) from deCODE Genetics were screened, followed by Bayesian co-localization and 2-step MR mediation analyses. Our findings revealed that ADHD was consistently associated with an increased risk of any stroke (AS), any ischemic stroke (AIS), and particularly large-artery atherosclerotic stroke (LAS; odds ratio = 1.455; 95% confidence interval: 1.077–1.967). Mediators screening showed genetic liability to ADHD was linked to elevated circulating matrix metallopeptidase-12 (MMP12; β = 0.141, *P* = .005), which conferred protection against AIS and LAS, potentially lowering LAS risk by 7.5%. In conclusion, this MR study suggests ADHD is a causal risk factor for ischemic stroke, especially LAS. Paradoxically, circulating MMP12 acts as a protective mediator, modestly reducing this risk by 7.5%. These findings require validation in future experimental and cohort studies.

## Graphical abstract

**Figure:**
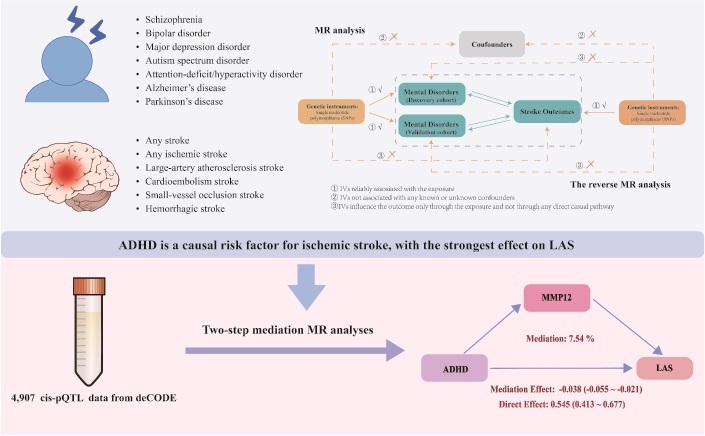

## 1. Introduction

Stroke, caused by vascular occlusion or rupture, is a leading global cause of death and disability.^[1,2]^ It results in over 7 million deaths annually and is projected to cause 10 million deaths each year by 2050, with economic costs exceeding 1.5 trillion USD.^[2]^ Stroke is classified into 2 main types: hemorrhagic, caused by vessel rupture, and ischemic, resulting from blocked blood flow and cerebral infarction.^[1,3]^ Hemorrhagic stroke (HS) often arises from aneurysms, arteriovenous malformations, or spontaneous intracerebral hemorrhage, whereas ischemic stroke (IS) has more complex etiologies. The Trial of Org 10,172 in Acute Stroke Treatment (TOAST) classification system categorizes IS into 5 subtypes: large-artery atherosclerosis stroke (LAS), caused by artery-to-artery thromboembolism from atherosclerotic lesions of the internal carotid artery or major intracranial arteries; cardioembolism stroke (CES), resulting from embolism originating in the heart; small-vessel occlusion stroke (SVS), due to occlusion of lenticulostriate arteries or vertebrobasilar perforators; stroke of other determined etiology; and stroke of undetermined etiology.^[3,4]^

Beyond conventional risk factors, mental disorders are increasingly recognized as potential contributors to stroke risk. Schizophrenia, bipolar disorder (BD), major depressive disorder (MDD), autism spectrum disorder (ASD), attention-deficit/hyperactivity disorder (ADHD), Alzheimer disease (AD), and Parkinson disease (PD) have all been implicated with an increased risk of stroke.^[5–9]^ However, despite consistent observational associations, the causal associations between mental disorders and stroke remain unclear due to confounding factors and limited mechanistic insight.^[10,11]^ Recent genome-wide association studies (GWAS) have provided new insights by identifying numerous risk loci. For example, the Psychiatric Genomics Consortium (PGC) identified 108 loci associated with schizophrenia, shedding light on synaptic function and neurodevelopmental pathways.^[12]^ Subsequent GWAS uncovered 33 risk loci for BD and approximately 44 loci for MDD, highlighting the roles of inflammatory pathways and neural plasticity in these disorders.^[13,14]^ In parallel, the MEGASTROKE meta-analysis (N = 521,612) identified novel stroke loci with shared vascular genetic architecture.^[15]^ Collectively, these large-scale GWAS suggest possible genetic susceptibility linking mental disorders and stroke, and provide critical genetic instruments for Mendelian randomization (MR) analyses, which use genetic variants as instrumental variables (IVs) to minimize confounding and reverse causality, thereby enabling causal inference beyond the limits of observational studies.^[16]^

This study employed bidirectional 2-sample MR analyses between 7 mental disorders and stroke subtypes. Analyses included discovery and validation cohorts of European ancestry, with MRlap and meta-analyses to derive pooled effect estimates. Two-step mediation MR identified circulating mediators, with Bayesian co-localization and GWAS meta-analysis confirming the findings. Collectively, we aimed to identify robust causal effects between mental disorders and stroke, as well as to detect potential mediators.

## 2. Methods

### 2.1. Study design

**Figure 1.**
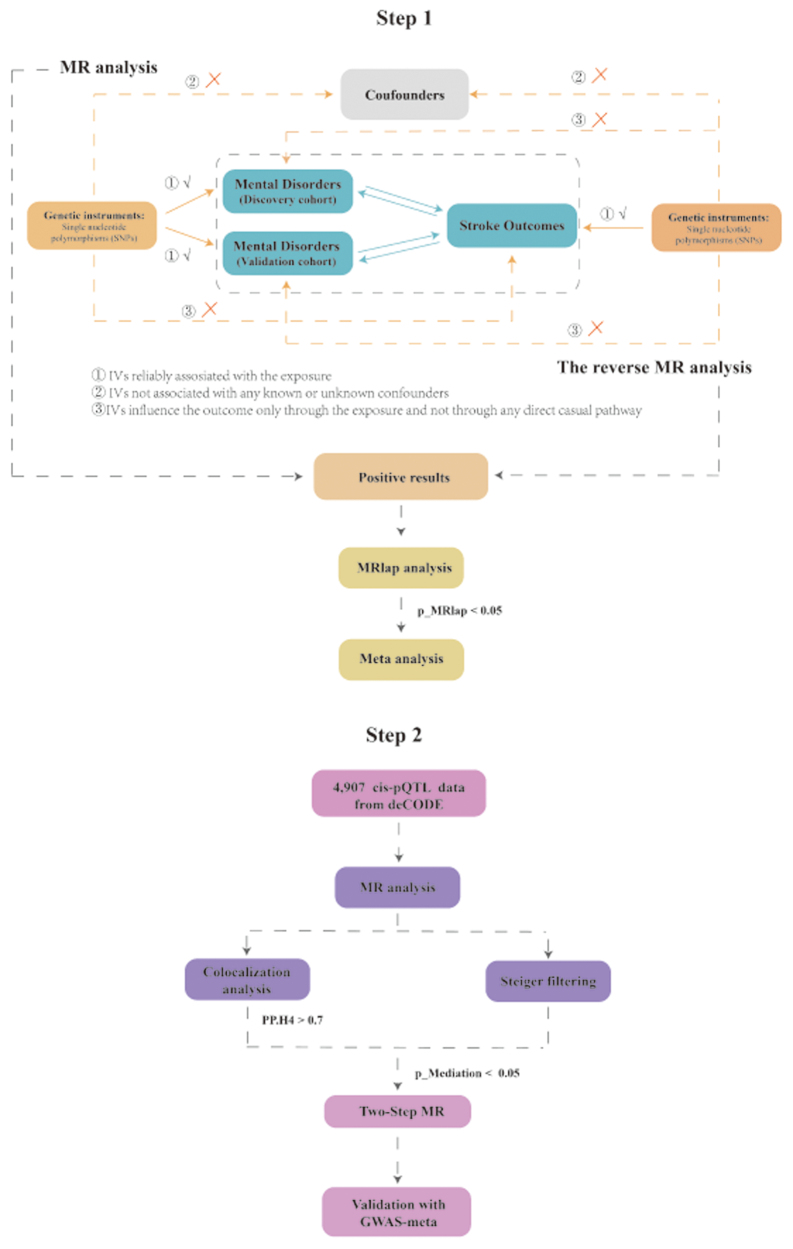
Overview of the 2-sample MR design investigating causal links between mental disorders and stroke subtypes. Step 1 used bidirectional MR analyses across discovery and validation cohorts. Step 2 applied MRlap correction and meta-analysis to refine estimates, and systematically screened 4907 plasma cis-pQTLs through MR and co-localization analyses. Candidate mediator proteins were further evaluated with 2-step MR, with GWAS meta-analysis providing final validation. MR, Mendelian randomization; GWAS, genome-wide association studies.

This study was conducted in 2 steps (Fig. 1). Step 1 employed bidirectional MR analyses to access causal effects between mental disorders and stroke. For robustness, 2 independent GWAS datasets were adopted as discovery and validation for each disorder. Step 2 further analyzed the significant associations identified in Step 1. Bayesian co-localization and 2-step mediation MR were applied to screen potential plasma protein mediators. Specifically, 4907 plasma cis-pQTLs from deCODE Genetics were tested for stroke outcomes using MR. After refining shared causal variants for significant proteins with Bayesian co-localization, a second MR analysis was performed to evaluate the mediating effects of prioritized proteins.

### 2.2. Data sources of mental disorders

For the discovery cohort, summary-level datasets for schizophrenia (N = 127,906), BD (N = 413,366), MDD (N = 500,199), ASD (N = 46,151), and ADHD (N = 55,374) patients were obtained from the PGC (https://pgc.unc.edu/). AD (N = 455,258) was obtained from the GWAS catalog (https://www.ebi.ac.uk/gwas/) and PD (N = 482,730) was obtained from the International Parkinson’s Disease Genomics Consortium (https://www.pdgenetics.org/).

For the validation cohort, summary-level datasets were obtained from the GWAS catalog, including schizophrenia (N = 48,253), BD (N = 456,348), MDD (N = 94,154), ASD (N = 54,976), ADHD (N = 53,293), AD (N = 63,926), and PD (N = 10,533) datasets. All discovery and validation datasets consisted of European ancestry (Supplementary Table 1).

Additionally, for ADHD specifically, we conducted a meta-analysis integrating data from both cohorts and an external dataset from Demontis et al (N = 225,534).^[17]^

### 2.3. Data sources of stroke

Summary-level datasets for stroke subtypes were obtained from the MEGASTROKE consortium (https://megastroke.org/),^[15]^ including: (i) any stroke (AS; N = 446,696), encompassing IS, HS, and strokes of undetermined etiology; (ii) any ischemic stroke (AIS; N = 440,328), and its subtypes: (iii) LAS (N = 150,765), (iv) CES (N = 211,763), and (v) SVS (N = 198,048). Additionally, the GWAS dataset GCST90043994 was incorporated for HS. All outcome datasets originated from European-ancestry cohorts (Supplementary Table 1).

### 2.4. Data sources of cis-pQTLs

Cis-pQTLs are genetic variants located within ± 1 Mb of the gene encoding the protein. In this study, cis-pQTLs data were obtained from deCODE Genetics (https://www.decode.com/), which includes whole-genome sequencing of 49,708 Icelandic individuals with comprehensive genotypic and phenotypic data. These data quantified 4907 unique plasma proteins in 35,559 individuals.^[18]^

### 2.5. Selection of IVs

IVs were single-nucleotide polymorphisms (SNPs) associated with the exposure of interest (Supplementary Tables 2,3,4 and 5). In this study, SNPs reaching genome-wide significance (*P* < 5 × 10^−8^) were used as IVs. When insufficient SNPs were available, relaxed thresholds were applied: *P* < 5 × 10^−6^ for ASD in the discovery cohort; for schizophrenia, BD, MDD, ASD, and ADHD in the validation cohort; and for stroke outcomes and deCODE plasma cis-pQTLs. *P* < 5 × 10^−5^ was used for HS. Linkage disequilibrium clumping was performed (r^2^ < 0.001, 10,000 kb) using European-ancestry reference data from the 1000 Genomes Project, and SNPs with F-statistics < 10 (β^2^/SE^2^) were excluded to reduce weak instrument bias.

### 2.6. MR analysis

MR analysis was conducted to evaluate causal associations between mental disorders and stroke, based on 3 core assumptions: IVs are reliably associated with the exposure; IVs are not associated with any known or unknown confounders; IVs influence the outcome only through exposure and not through any direct causal pathway.^[16]^ The primary method was inverse-variance weighted (IVW) regression with multiplicative random effects to account for heterogeneity.^[19]^ Horizontal pleiotropy was assessed using the MR-Egger intercept,^[20]^ and outliers were detected and removed using MR-PRESSO.^[21]^ Heterogeneity was assessed by Cochran Q-test, and the robustness was examined through leave-one-out sensitivity analysis.

Two-step MR was used to investigate potential mediation by 4907 cis-pQTLs, decomposing the total effect (β_0_) into exposure-to-mediator (β_1_) and mediator-to-outcome (β_2_) effects. Mediation effects (β_1_ × β_2_) and the proportion mediated (β_1_ × β_2_/ β_0_) were estimated when both associations were significant.^[22]^

All analyses were conducted in using the *TwoSampleMR* and *MR-PRESSO* packages (R v 4.4.0).

### 2.7. MRlap analysis

Given that all datasets were from European ancestry, MRlap analysis was applied to account for sample overlap bias by employing cross-trait linkage disequilibrium score regression to correct MR estimates.^[23]^ Concordance between the corrected and original estimates confirmed the reliability of the IVW results. All analyses were performed using the *MRlap* package (R v 4.4.0).

### 2.8. Steiger filtering analysis

To minimize potential reverse causality for cis-pQTLs, Steiger filtering was conducted using the *TwoSampleMR* R package. This method compares IVs for the exposure and outcome byquantifying R^2^: $R^{2}\left(XY\right)=\frac{\left(Cov{\left(X,\;Y\right)}^{2}\right)}{\left(Var\left(X\right)*\;Var\left(Y\right)\right)}$,^[24]^ where higher IV-exposure R^2^ values indicate that the causal direction is more likely from exposure to outcome, thereby supporting causal inference.

### 2.9. Co-localization analysis

Bayesian co-localization analysis was employed to evaluate whether traits share causal variants at specific genomic loci. Posterior probabilities were calculated for 5 competing hypotheses, with Hypothesis 4 (PPH4) indicating that both traits are associated with the same causal variant within a locus.^[23]^ PPH4 posterior probability > 0.7 was considered strong evidence for shared causal variants between traits. The *coloc* package (R v 4.4.0) was used to perform the co-localization analysis.

### 2.10. Meta-analysis

Meta-analysis was performed in both steps to minimize bias and obtain pooled effect estimates. In Step 1, significant MR estimates were combined with external GWAS datasets using the R package *meta*. Either common-effect or random-effects models were applied depending on heterogeneity (I^2^ > 50%). In Step 2, summary-level GWAS meta-analysis was conducted with METAL, employing sample-size-weighted common-effect models optimized for unbalanced case-control designs. Built-in heterogeneity testing in METAL (University of Michigan Center for Statistical Genetics) was used to ensure consistency of effects across cohorts.^[25]^ The meta-analyzed GWAS dataset was subsequently used to validate the mediation analyses.

## 3. Results

### 3.1. ADHD increases stroke risk, whereas AD is a protective factor for CES

**Figure 2.**
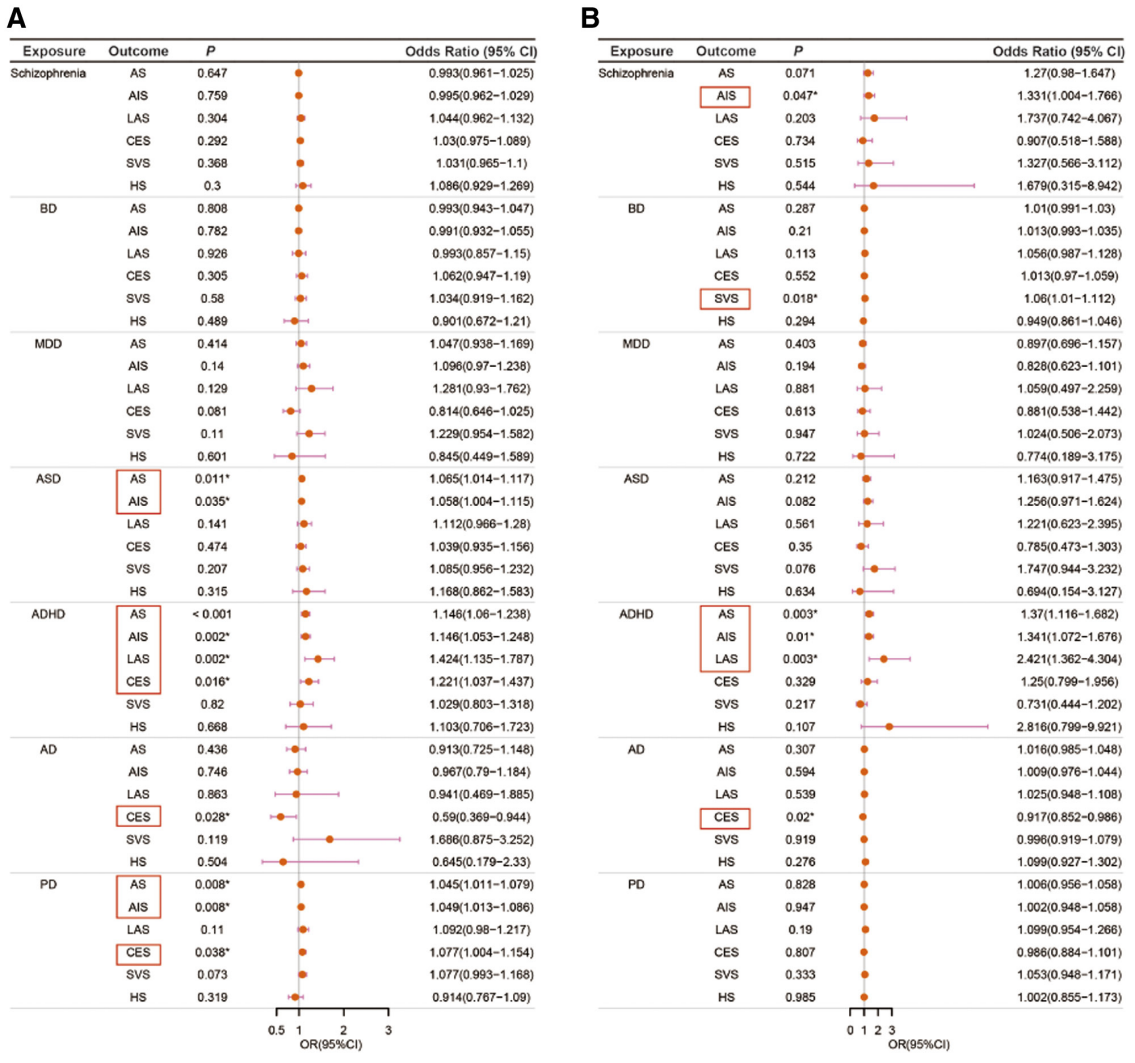
MR results for mental disorders on stroke subtypes. Forest plots of Mendelian randomization estimate for mental disorders on stroke subtypes, including (A) discovery cohort and (B) validation cohort. Each row represents an exposure-outcome pair, with OR and 95% CI shown as points and horizontal lines. Red boxes highlight outcomes with statistically significant associations. MR, Mendelian randomization; OR, odds ratio; CI, confidence interval.

Across both the discovery and validation cohorts, ADHD was consistently associated with increased risks of AS, AIS, and LAS. In the discovery cohort, ADHD was linked to higher risks of AS (odds ratio [OR] = 1.146, 95% confidence interval [CI]: 1.060–1.238, *P < *.001), AIS (OR = 1.146, 95% CI: 1.053–1.248, *P* = .002), and LAS (OR = 1.424, 95% CI: 1.135–1.787, *P* = .002) (Fig. 2A; Supplementary Table 6). These associations were reinforced in the validation cohort, with elevated risks for AS (OR = 1.370, 95% CI: 1.116–1.682, *P* = .003), AIS (OR = 1.341, 95% CI: 1.072–1.676, *P* = .01), and LAS (OR = 2.421, 95% CI: 1.362–4.304, *P* = .003) (Fig. 2B; Supplementary Table 7). In contrast, AD exhibited a consistent protective effect against CES, evident in both the discovery (OR = 0.590, 95% CI: 0.369–0.944, *P* = .03) (Fig. 2A; Supplementary Table 6) and validation cohorts (OR = 0.917, 95% CI: 0.852–0.986, *P* = .02) (Fig. 2B; Supplementary Table 7).

Sensitivity analyses showed no heterogeneity and pleiotropy in the associations of ADHD and AD with their related stroke subtypes (Table 1). Leave-one-out analyses further confirmed the robustness of these findings (Supplementary Figures 1,2,3 and 4).

**Table 1:** Sensitivity analyses for pleiotropy and heterogeneity in MR estimates of mental disorders and stroke subtypes.

Exposure	Outcome	MR Egger intercept	MR Egger *P*	Cochran Q *P*
ADHD (Discovery)	AS	0.009	0.587	.590
	AIS	0.016	0.357	.710
	LAS	−0.038	0.433	.320
ADHD (Validation)	AS	0.016	0.476	.492
	AIS	0.006	0.812	.556
	LAS	−0.027	0.672	.322
AD (Discovery)	CES	0.006	0.555	.126
AD (Validation)		−0.007	0.502	.577
SVS (Discovery)	ASD	0.004	0.677	.637
SVS (Validation)		0.002	0.200	.567

AD = Alzheimer disease, ADHD = attention-deficit/hyperactivity disorder, AIS = any ischemic stroke, AS = any stroke, ASD = autism spectrum disorder, CES = cardioembolism stroke, LAS = large-artery atherosclerosis stroke, MR = Mendelian randomization, SVS = small-vessel occlusion stroke.

### 3.2. SVS serves as a risk factor for ASD

**Figure 3.**
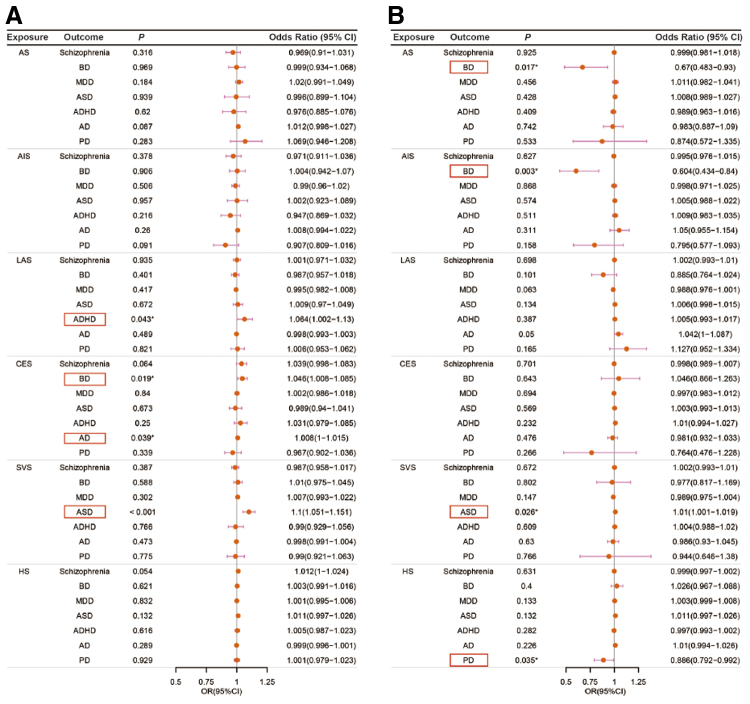
MR results for stroke subtypes on mental disorders. Forest plots of Mendelian randomization estimate for stroke subtypes on mental disorders, including (A) discovery cohort and (B) validation cohort. Each row represents an exposure-outcome pair, with OR and 95% CI shown as points and horizontal lines. Red boxes highlight outcomes with statistically significant associations. MR, Mendelian randomization; OR, odds ratio; CI, confidence interval.

Across both discovery and validation cohorts, SVS was consistently associated with increased ASD risk. In the discovery cohort, SVS elevated ASD risk (OR = 1.100, 95% CI: 1.051–1.151, *P* < .001) (Fig. 3A; Supplementary Table 8). This association was replicated in the validation cohort (OR = 1.010, 95% CI: 1.001–1.019, *P* = .03) (Fig. 3B; Supplementary Table 9).

Sensitivity analyses showed no heterogeneity and pleiotropy in the associations of SVS and ASD (Table 1). Leave-one-out analyses further confirmed the robustness of these findings (Supplementary Figures 5, 6).

### 3.3. MRlap correction confirms robustness of ADHD-stroke associations

MRlap correction was applied to address potential sample overlap bias.^[23]^ After correction, the protective effects of AD on CES and the detrimental effect of SVS on ASD were no longer statistically significant, whereas the detrimental effects of ADHD on stroke subtypes remained significant, supporting their robustness (Table 2; Supplementary Table 10).

**Table 2:** MRlap-corrected estimates for associations between mental disorders and stroke subtypes.

Exposure	Outcome	Beta (SE)	*P*
ADHD (Discovery)	AS	0.074 (0.035)	.034*
	AIS	0.072 (0.028)	.010*
	LAS	0.118 (0.054)	.029*
ADHD (Validation)	AS	0.064 (0.031)	.041*
	AIS	0.063 (0.025)	.013*
	LAS	0.143 (0.048)	.003*
AD (Discovery)	CES	−0.031 (0.027)	.252
AD (Validation)		−0.012 (0.010)	.210
SVS (Discovery)	ASD	0.374 (70,443.420)	1.000
SVS (Validation)		0.108 (114.736)	.999

AD = Alzheimer disease, ADHD = attention-deficit/hyperactivity disorder, AIS = any ischemic stroke, AS = any stroke, ASD = autism spectrum disorder, CES = cardioembolism stroke, LAS = large-artery atherosclerosis stroke, SE = standard error, SVS = small-vessel occlusion stroke.

### 3.4. Meta-analysis estimating the effects of ADHD on stroke subtypes

**Figure 4.**
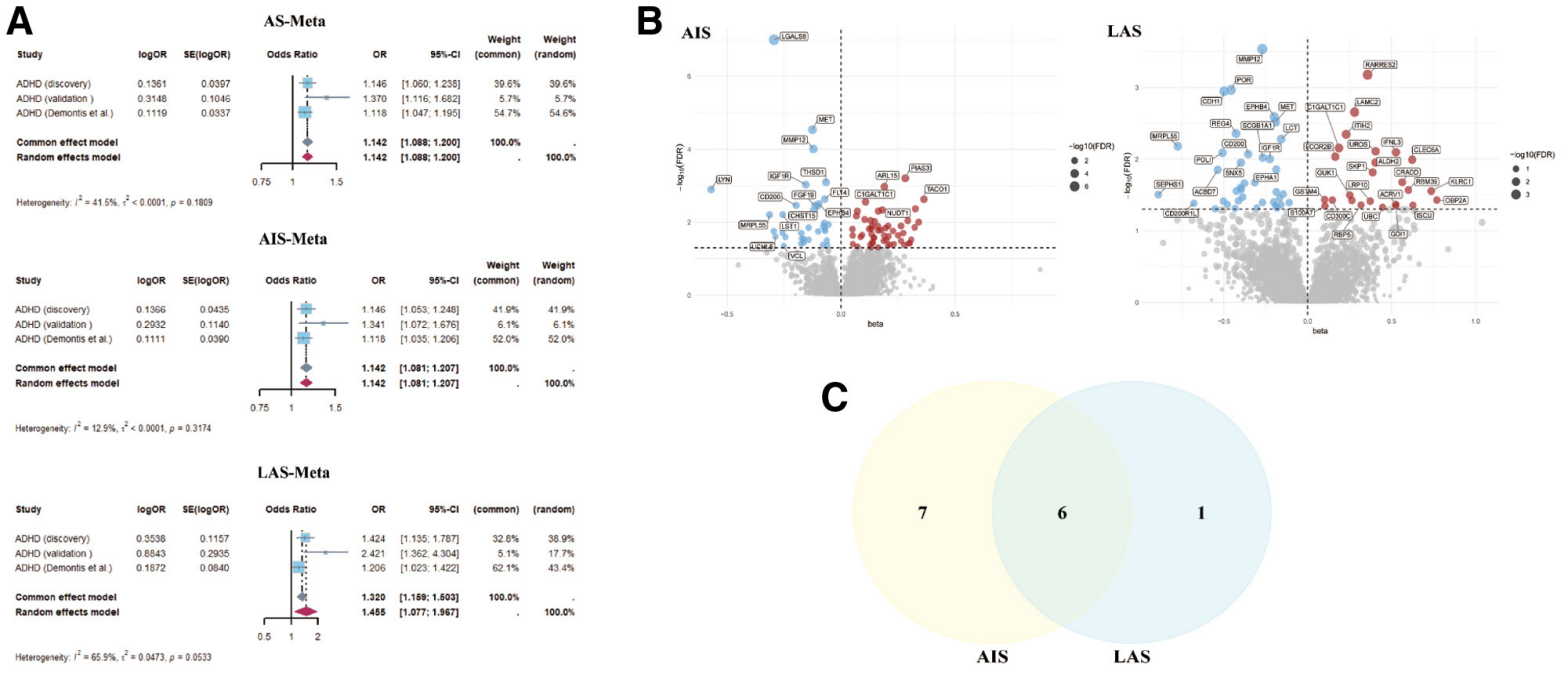
Meta-analysis and protein screening results. (A) Forest plots of meta-analyses evaluating the effect of ADHD on stroke subtypes (AS, AIS, LAS). Points represent OR with 95% CI. Heterogeneity statistics (I^2^ and P values) are shown below each panel to indicate consistency across datasets; higher I^2^ values reflect greater heterogeneity. (B) Volcano plots of MR analyses screening plasma proteins associated with AIS and LAS after FDR correction (FDR < 0.05). Red points represent proteins upregulated by ADHD, and blue points represent proteins downregulated; point size reflects statistical significance. (C) Venn diagram showing overlap of proteins associated with AIS and LAS after co-localization analysis. MR, Mendelian randomization; ADHD, attention-deficit/hyperactivity disorder; AS, any stroke; AIS, any ischemic stroke; LAS, large-artery atherosclerosis stroke; OR, odds ratio; CI, confidence interval; FDR, false discovery rate.

Although ADHD showed consistent associations with increased risks of AS, AIS, and LAS, effect sizes varied across subtypes, particularly for LAS. To obtain more reliable estimates, we performed a meta-analysis of ADHD results, incorporating the ADHD dataset reported by Demontis et al., which also demonstrated significant associations with the same stroke subtypes (AS: OR = 1.118, 95% CI: 1.047–1.195, *P* < .001; AIS: OR = 1.118, 95% CI: 1.035–1.206, *P* = .004; LAS: OR = 1.206, 95% CI: 1.023–1.422, *P* = .03; Supplementary Figure 7). The final pooled estimates indicating significant associations of ADHD on AS (OR = 1.142, 95% CI: 1.088–1.200), AIS (OR = 1.142, 95% CI: 1.081–1.207), with the most pronounced effect seen in LAS (OR = 1.455, 95% CI: 1.077–1.967; random-effects model given I^2^ > 50%) (Fig. 4A; Supplementary Figure 8). Notably, the identical effect sizes for AS and AIS, coupled with the absence of significant associations with HS, initially indicated that the elevated stroke risk is primarily driven by ischemic events. Consequently, our subsequent analyses focused on IS and its subtypes, with a particular emphasis on LAS, which demonstrated the most prominent effect.

### 3.5. Screening of plasma proteins associated with the AIS and LAS

IS accounts for 65.3% of all stroke cases, with high incidence (92.4/100,000) and disability rates (44.2/100,000).^[26]^ Among IS subtypes, LAS is the most prevalent and clinically significant, driven by large-artery plaque accumulation that causes extensive cerebral ischemia, high recurrence risk, and poor prognosis.^[27]^ However, the pathogenesis of LAS remains incompletely understood.

To explore potential circulating protein mediators of IS subtypes, cis-pQTLs of 4907 plasma proteins from deCODE Genetics were systematically screened to identify mediators of AIS and LAS. MR analysis with false discovery rate (FDR) correction (FDR < .05) identified 88 AIS-associated and 63 LAS-associated proteins (Fig. 4B; Supplementary Table 11). Bayesian Co-localization analyses confirmed 13 proteins sharing causal variants with AIS and 7 with LAS (Table 3; Supplementary Table 12; Supplementary Figures 9,10,11,12,13,14,15,16,17,18,19,20,21,22,23,24,25,26,27 and ,28). Notably, 6 LAS-co-localized proteins also associated with AIS, except ubiquitin C (Fig. 4C; Supplementary Table 12).

**Table 3:** Mediation MR results of plasma proteins co-localized with ADHD effects on IS (AIS, LAS).

Exposure	Outcome	Protein	PP.H4.abf	Mediation effect	Direct effect	Mediation proportion	*P*
ADHD	AIS	KNG1	0.738	−0.002 (−0.210–0.207)	0.138 (0.095–0.182)	−0.012 (−1540–1.516)	.526
		MET	1.000	−0.003 (−0.007–0.002)	0.139 (0.096–0.183)	−0.020 (−0.054–0.014)	.554
		IGF1R	1.000	0.004 (−0.004–0.012)	0.132 (0.088–0.177)	0.030 (−0.029–0.090)	.609
		CHST15	1.000	−0.009 (−0.014–0.004)	0.146 (0.102–0.189)	−0.066 (-0.105–0.027)	.090
		**MMP12**	**0.989**	−**0.015** (−**0.021–0.008**)	**0.151 (0.107–0.195**)	−**0.107** (−**0.153–**−**0.061**)	.020*
		LGALS8	1.000	−0.003 (−0.016–0.010)	0.140 (0.094–0.185)	−0.023 (−0.116–0.070)	.808
		JAG1	1.000	0.008 (−0.001–0.017)	0.128 (0.084–0.173)	0.061 (−0.005–0.127)	.357
		CD200	1.000	−0.003 (−0.011–0.005)	0.140 (0.095–0.184)	−0.023 (−0.116–0.070)	.707
		LIFR	1.000	−0.002 (−0.210–0.207)	0.136 (0.092–0.179)	0.008 (−0.026–0.041)	.817
		EPHB4	1.000	−0.003 (−0.011–0.005)	0.140 (0.096–0.183)	−0.024 (−0.051–0.004)	.389
		L1CAM	1.000	0.003 (−0.003–0.009)	0.134 (0.090–0.177)	0.022 (−0.022–0.067)	.619
		TPST2	1.000	0.012 (0.003–0.020)	0.125 (0.080–0.169)	0.087 (0.025–0.148)	.162
		IL2RB	0.999	−0.004 (−0.011–0.003)	0.142 (0.098–0.186)	−0.042 (−0.099–0.016)	.471
	LAS	MET	0.818	−0.004 (−0.011–0.003)	0.358 (0.242–0.474)	−0.012 (−0.032–0.008)	.557
		IGF1R	0.8181	−0.001 (−0.011–0.008)	0.355 (0.239–0.471)	−0.004 (−0.031–0.024)	.889
		**MMP12**	**0.987429947**	−**0.031** (−**0.043–0.018**)	**0.385 (0.268–0.501**)	−**0.087** (−**0.123–0.051**)	.015*
		LGALS8	0.818142013	0 (−0.015–0.015)	0.354 (0.237–0.471)	−0.001 (−0.044–0.042)	.981
		CD200	0.818096086	−0.005 (−0.017–0.008)	0.358 (0.242–0.475)	−0.013 (−0.049–0.022)	.712
		EPHB4	0.818117646	−0.007 (−0.014–0.001)	0.360 (0.244–0.476)	−0.01 9 (-0.040–0.003)	.386
		UBC	0.796990582	0.013 (−0.007–0.034)	0.340 (0.223–0.458)	0.038 (−0.020–0.340)	.511

ADHD = attention-deficit/hyperactivity disorder, AIS = any ischemic stroke, IS = ischemic stroke, LAS = large-artery atherosclerosis stroke, MR = Mendelian randomization.

### 3.6. No reverse causality in Steiger filtering analysis

After identifying causal variants through co-localization, Steiger filtering was applied to the prioritized proteins to assess reverse causality. All IVs indicated the correct direction of effect, supporting protein-to-stroke causal associations (Supplementary Table 13).

### 3.7. MMP12 serves as a protective mediator of the ADHD-IS associations

**Figure 5.**
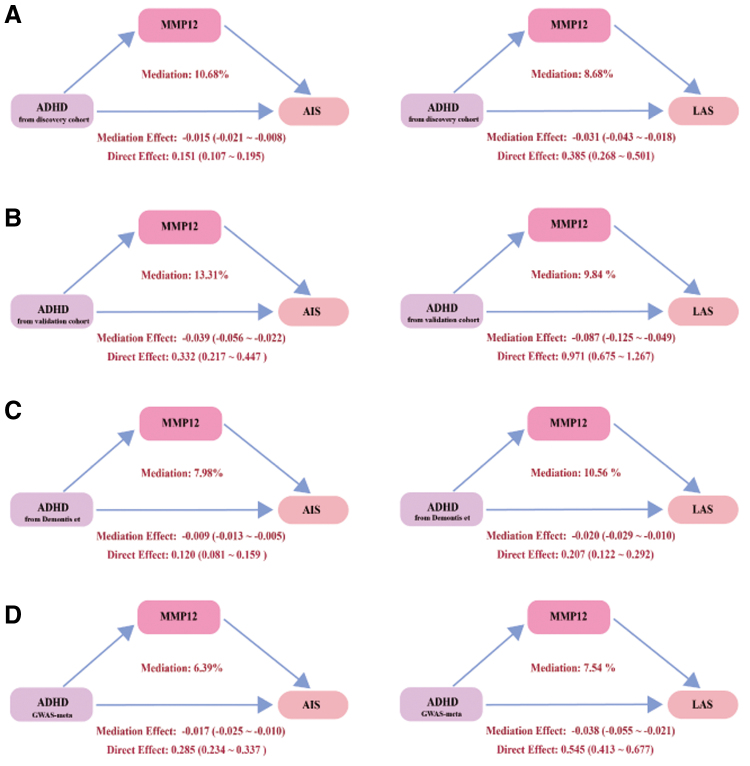
Mediation of ADHD effects on IS (AIS, LAS) via MMP12. Two-step MR results show MMP12 as a mediator of ADHD effects on AIS (left) and LAS (right). Mediation proportions, mediation effects, and direct effects are reported for (A) discovery cohort, (B) validation cohort, (C) Demontis et al dataset, and (D) GWAS meta-analysis dataset. MR, Mendelian randomization; ADHD, attention-deficit/hyperactivity disorder; IS, ischemic stroke; AIS, any ischemic stroke; LAS, large-artery atherosclerosis stroke; MMP12, matrix metallopeptidase-12.

In the 2-step MR analysis of the discovery cohort, circulating matrix metallopeptidase-12 (MMP12) emerged as the primary candidate protein demonstrating significant protective mediation effects on both AIS (*P* = .02, mediation effect = -0.015) and LAS (*P* = .02, mediation effect = -0.031). Validation analyses corroborated these findings, suggesting that genetically predicted ADHD-associated MMP12 upregulation was linked to protective effects against AIS (*P* = .02, mediation effect = -0.039) and LAS (*P* = .02, mediation effect = -0.087). Consistent protective trends were also observed in the external ADHD dataset reported by Demontis et al. (AIS: *P* = .04, mediation effect = -0.009; LAS: *P* = .04, mediation effect = -0.020) (Fig. 5; Supplementary Tables 14, 15).

To obtain more reliable effect estimates, a GWAS meta-analysis of the 3 ADHD datasets was performed using METAL with sample-size weighting. The pooled results indicated that genetic liability to ADHD was significantly associated with elevated circulating MMP12 levels (β = 0.141, *P* = .005). Furthermore, this systemic upregulation acted as a potential protective mediator, modestly attenuating the total risk of AIS and LAS by 6.39% and 7.54%, respectively (Fig. 5; Supplementary Table 15).

## 4. Discussion

In our bidirectional 2-step MR analysis, we systematically evaluated the causal effects of 7 mental disorders on stroke and its subtypes, and then explored plasma protein mediators. Across discovery, validation, and MRlap correction, only ADHD showed consistent and robust causal detrimental effects on IS, particularly LAS. The final pooled estimate indicated that genetic liability to ADHD increased LAS risk by approximately 45% (OR = 1.455, 95%CI: 1.077–1.967).

Through the screening of 4907 cis-pQTLs, we identified a small set of proteins that shared causal variants with LAS. Among them, MMP12 emerged as the primary mediator. Genetically predicted ADHD was associated with higher circulating MMP12 concentrations, which in turn were causally linked to lower LAS risk, attenuating the total ADHD-attributable risk by about 7.5%.

ADHD, affecting 5.29% of youth, is increasingly recognized as an independent cerebrovascular risk phenotype rather than merely a stroke sequela.^[28,29]^ Although concerns were initially raised regarding the safety of ADHD medications, pharmacoepidemiological studies have ruled them out as major cardiovascular risk factors.^[30]^ Phenome-wide association study based on the Estonian Biobank has shown that higher polygenic risk scores for ADHD are significantly associated with a wide range of cardiovascular diseases (CVDs), including type 2 diabetes, coronary artery disease, and stroke.^[31]^ Some MR studies also suggest recently that ADHD itself is a risk factor for CVDs.^[32–34]^

A core question lies in how ADHD, a neurodevelopmental disorder defined by altered dopaminergic and noradrenergic circuits, can exert long-term, physically destructive effects on systemic vasculature. Our study reveals that ADHD has the strongest causal effect on LAS, rather than on CES or SVS, offering a new avenue. Given that atherosclerosis primarily arises from persistent metabolic dysregulation and chronic inflammation, it is highly plausible that ADHD genetic liability increases LAS risk by gradually damaging and remodeling the vascular endothelium and intimal matrix over decades.^[35,36]^

Metabolic dysregulation is a recurring theme in ADHD pathophysiology. Due to dopamine system abnormalities triggering a “reward deficiency syndrome,”^[28,37]^ ADHD patients often exhibit a preference for immediate gratification, and tend to consume high-sugar, high-fat, high-calorie foods to processed foods to temporarily stimulate dopamine release.^[38,39]^ This predisposes them to obesity, dyslipidemia, and poor glycemic control. Furthermore, the Dutch Lifelines cohort study identifies a genetic nexus: higher ADHD polygenic scores are positively correlate with adverse lipid profiles, hypertension, and compromised liver function.^[40]^ In other words, the genetic liability to ADHD itself carries a metabolic predisposition, making patients prone to developing a pro-atherosclerotic state characterized by hyperlipidemia and hyperglycemia.

Furthermore, chronic low-grade inflammation has been highlighted as associated with ADHD genetic architecture. Numerous studies have confirmed that ADHD correlates with abnormal circulating levels of cytokine genes (IL-1 family, IL-6, TNF-α, IL-16).^[41,42]^ Proteomic clustering analysis has even identified a “higher inflammatory potential” subtype of adult ADHD, characterized by more severe chronic stress, higher overall clinical severity, and persistent hyperactivation of signaling pathways, such as NF-κB, chemokine, and IL-17.^[43]^ This lifelong inflammatory phenotype provides an ideal catalyst for LAS. In vascular biology, chronic systemic inflammation drives endothelial dysfunction, and upregulates adhesion molecules like VCAM-1 and ICAM-1, which triggers sustained monocyte recruitment and migration into the intima.^[36,37,44]^ Once there, these cells differentiate into macrophages and transform into lipid-laden foam cells. This process initiates a vicious cycle: the continuous secretion of inflammatory mediators and matrix metalloproteinases expands the lipid core while thinning the fibrous cap, eventually predisposed to plaque rupture.^[36,44]^

In our mediation analysis, MMP12 was identified as the primary circulating protein mediator, exerting a protective effect and accounting for a 7.5% reduction in overall LAS risk. MMP12, also known as macrophage metalloelastase, is produced almost exclusively by activated macrophages and efficiently degrades key extracellular matrix (ECM) components, including elastin, fibronectin, laminin, and type IV collagen.^[45]^ Traditionally, MMP12 has been viewed as a strongly pathogenic factor in atherosclerosis: once macrophages infiltrate the arterial wall and form a lipid core, they locally overexpress and secrete MMP12. High focal concentrations of MMP12 rapidly degrade the elastin-rich fibrous cap, leading to cap thinning and structural instability.^[46–48]^ The resulting ECM breakdown further facilitates monocyte and inflammatory cell recruitment, establishing a self-perpetuating positive feedback loop.

However, large-scale genomic and proteomic studies, align with our findings, indicating that higher circulating MMP12 levels exert an anti-stroke effect. MR analyses based on MEGASTROKE have quantified this association: for each standard deviation decrease in genetically predicted serum MMP12, the risk of any IS increases (OR ≈ 0.90), with an even stronger effect for LAS (OR ≈ 0.71).^[49,50]^ Extensive phenotypic MR analyses of plasma proteomics similarly identified MMP12 as a potential protective mediator against IS.^[50,51]^ Notably, Mahdessian et al revealed a significant difference in plasma MMP12 levels between clinical observations and genetic effects. While clinical data showed that plasma MMP12 concentrations correlate positively with carotid intima-media thickness (cIMT) and plaque area, at the genetic level, the LAS-associated risk loci identified in the 11q22.3 region (rs660599, rs499459) associated with LAS corresponded to lower plasma MMP12 levels despite increasing cIMT.^[52]^ This genetic evidence supports our findings that circulating MMP12 levels may be lower at baseline in individuals with high LAS risk. However, current clinical studies consistently report elevated plasma MMP12 in atherosclerotic patients, with symptomatic patients exhibiting high MMP12 expression and inflammatory infiltration at lesion sites.^[52,53]^ Furthermore, loss-of-function experiments confirm that MMP12 gene knockout reduces lesion burden.^[52,54,55]^ Overall, interpretation of MMP12’s biological effects warrants caution. First, circulating MMP12 and locally expressed MMP12 may exert distinct functions. Second, in the LAS context, the genetic effect on circulating MMP12 diverges from clinically observed associations. This discrepancy may stem from differing disease progression stages, interference from other inflammatory pathways, or complex feedback regulatory mechanisms.

Although the 7.5% mediating effect of circulating MMP12 was statistically significant, its biological impact was modest. We hypothesize that this limited effect arises because MMP12 represents only a minor node within the complex regulatory network governing the transition from neurodevelopmental traits to vascular pathology. Its primary effect also contradicts ADHD, partially counteracting the pro-atherosclerotic environment associated with genetic liability to ADHD. Therefore, it should be regarded as a compensatory mechanism, and the primary drivers of overall risk remain to be identified.

## 5. Limitations

Several limitations must be acknowledged. First, although MR analysis provides causal evidence at the genetic level, it inherently cannot directly observe specific molecular or cellular biological processes. Consequently, the interpretation of its results must be confined to the level of causal effects. And due to the lack of subsequent functional experimental validation, our research findings should be regarded as “hypothesis generation” rather than “mechanism confirmation.” Second, the MMP gene cluster exhibits high polymorphism, which implies that captured genetic signals may incorporate interference from adjacent genes. This makes it challenging to fully exclude non-MMP12-specific overlapping effects. Furthermore, while MMP12’s mediating role is statistically significant, it accounts for only 7.5% of the risk reduction. This limited protective effect may stem from its biological properties as a counter-regulatory factor, strongly suggesting that more dominant pathogenic mechanisms remain unidentified in the ADHD-LAS pathway. Fourth, this study primarily relies on plasma cis-pQTLs data to infer vascular mediators, yet circulating protein levels may not accurately reflect local expression or enzymatic activity within atherosclerotic plaques or cerebral arteries. As discussed, the biological effects of the MMP12 may differ markedly between the circulatory system and local lesions, and divergence exists between genetic effects and clinical reality, necessitating caution in interpreting results. Finally, all analyses were based on GWAS data from European populations. While this approach reduces population stratification bias and enhances internal robustness of results, it significantly constrains the generalizability of conclusions. Caution is warranted when extrapolating findings to other populations with distinct genetic backgrounds and environmental risk factors.

## 6. Conclusion

This study provides robust genetic evidence that ADHD increases the risk of ischemic stroke, primarily LAS. Paradoxically, circulating MMP12 is implicated as a protective mediator, modestly attenuating this risk by 7.5%. Given the lack of experimental validation, these findings warrant future experimental and cohort studies to confirm.

## Acknowledgments

The authors sincerely thank the builders and participants of the public databases, including but not limited to the Psychiatric Genomics Consortium, the MEGASTROKE Consortium, the GWAS catalog, the International Parkinson’s Disease Genomics Consortium, and the deCODE Genetics.

## Author contributions

**Conceptualization:** Hang Ji.

**Data curation:** Linhao Cong, Hang Ji, Hailan Yang.

**Formal analysis:** Linhao Cong, Hang Ji, Guicheng Kuang.

**Investigation:** Linhao Cong, Hang Ji, Hailan Yang, Guicheng Kuang, Haitao Liu.

**Project administration:** Hailan Yang, Haitao Liu.

**Supervision:** Haogeng Sun, Yi Liu.

**Writing – original draft:** Linhao Cong, Hang Ji, Haitao Liu.

**Writing – review & editing:** Haogeng Sun, Yi Liu.









**Figure:**
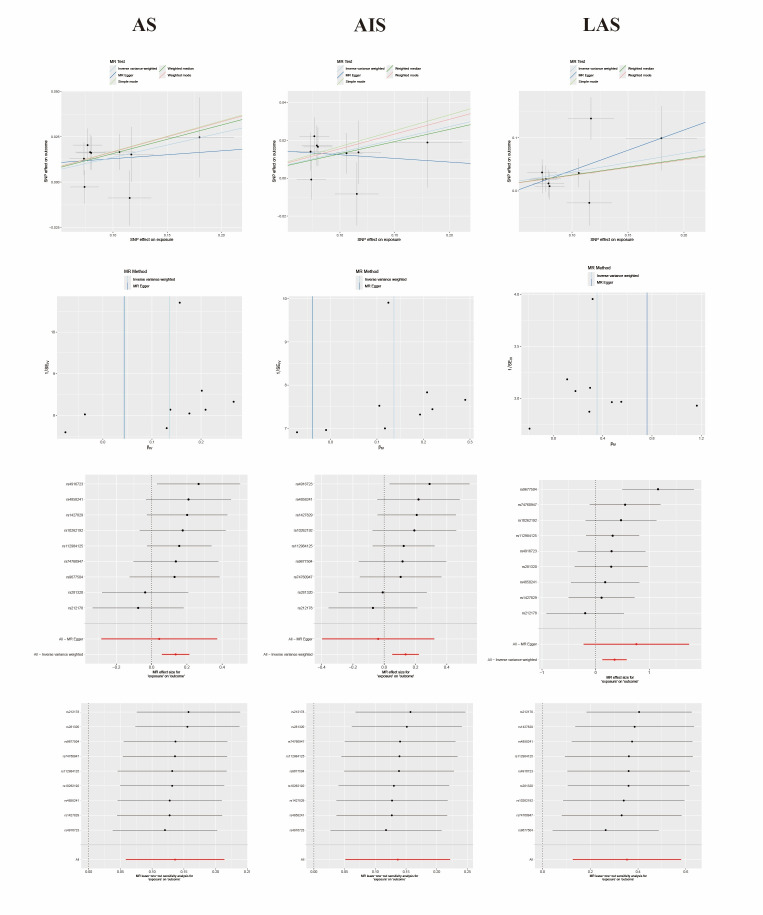






**Figure:**
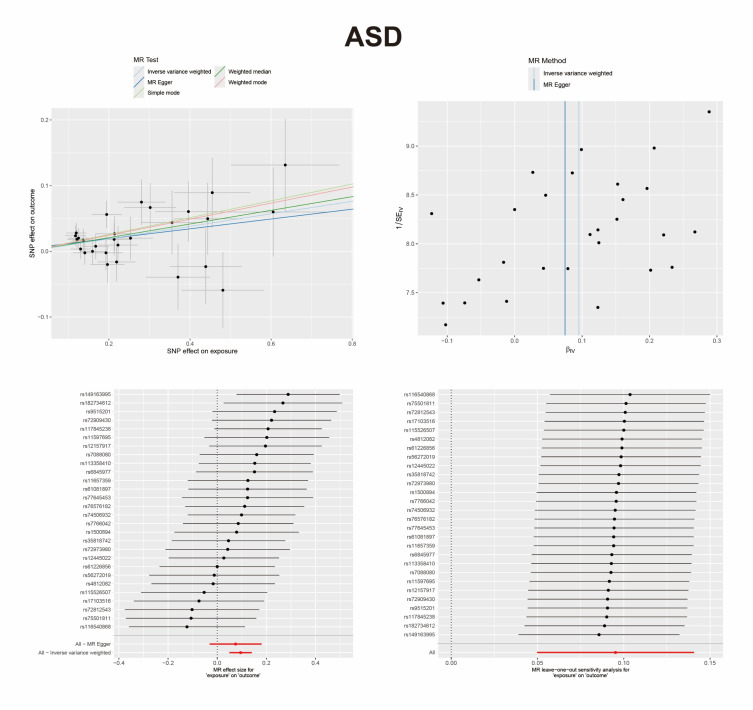




**Figure:**
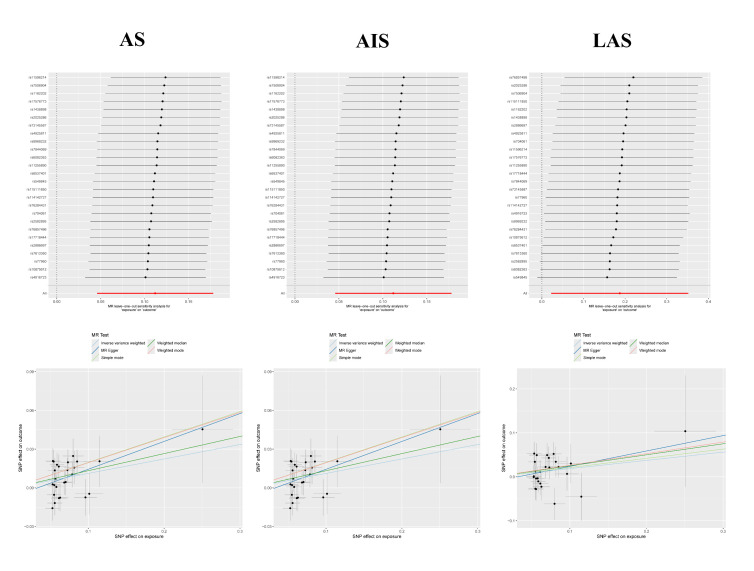


**Figure:**
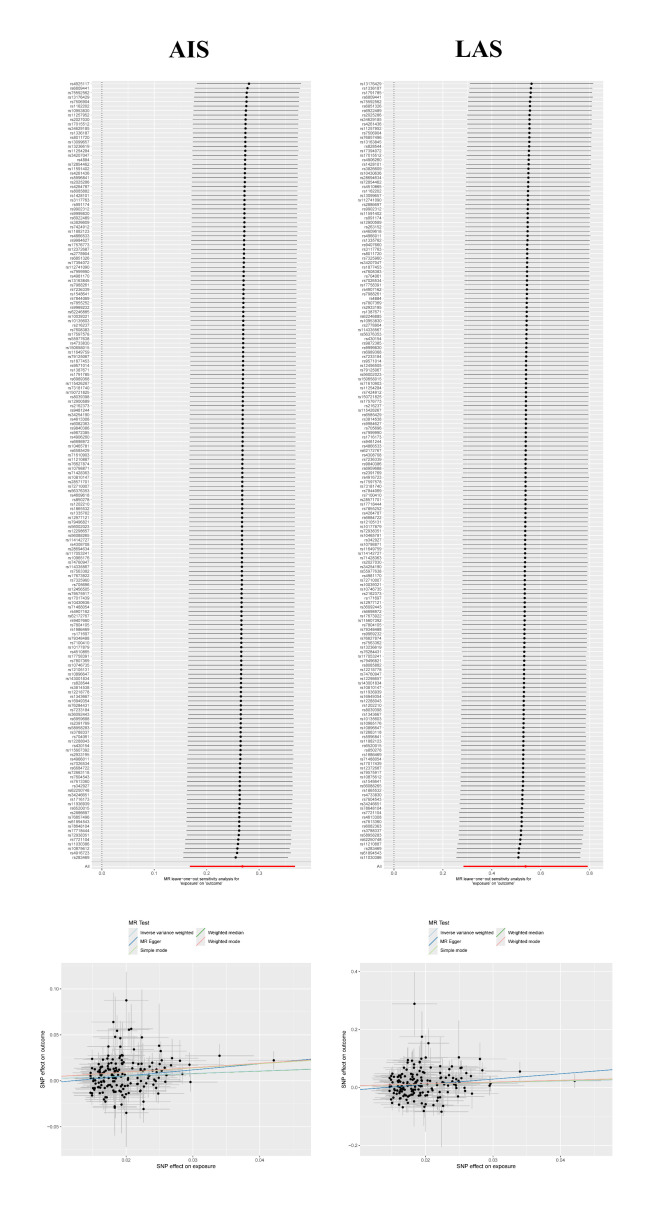






**Figure:**
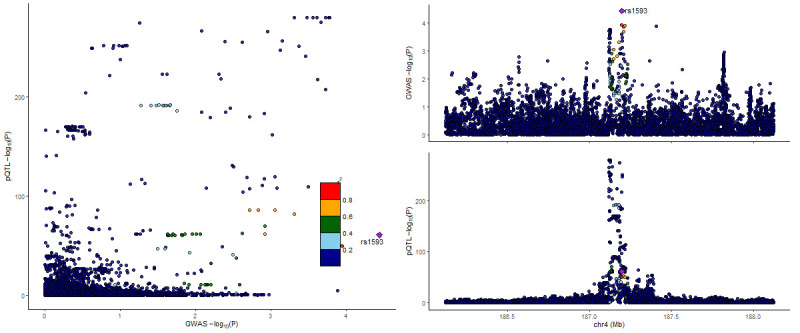














**Figure:**
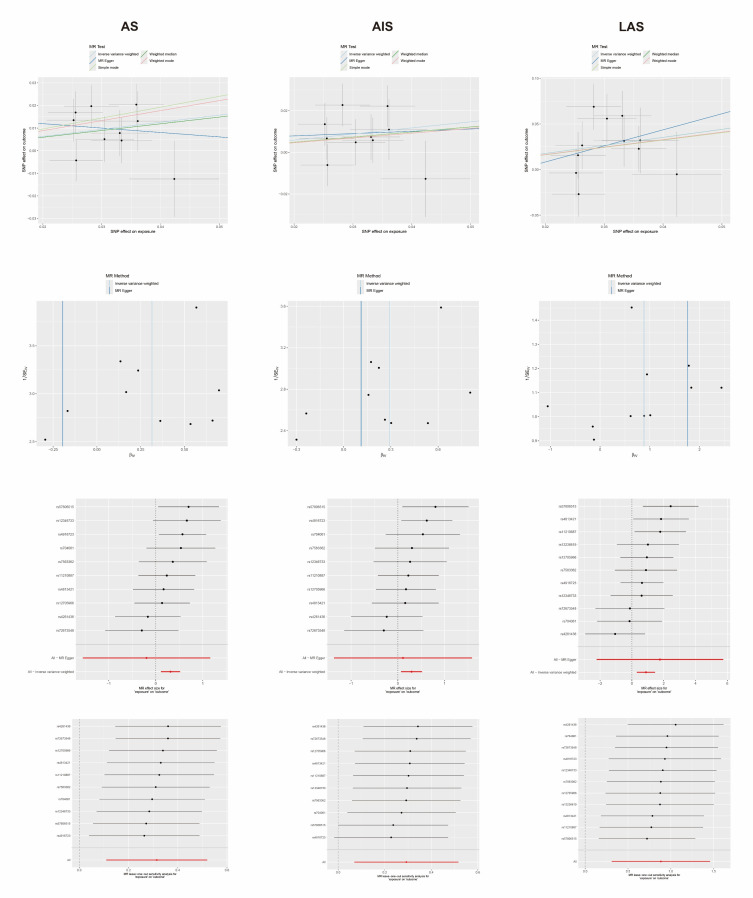


**Figure:**
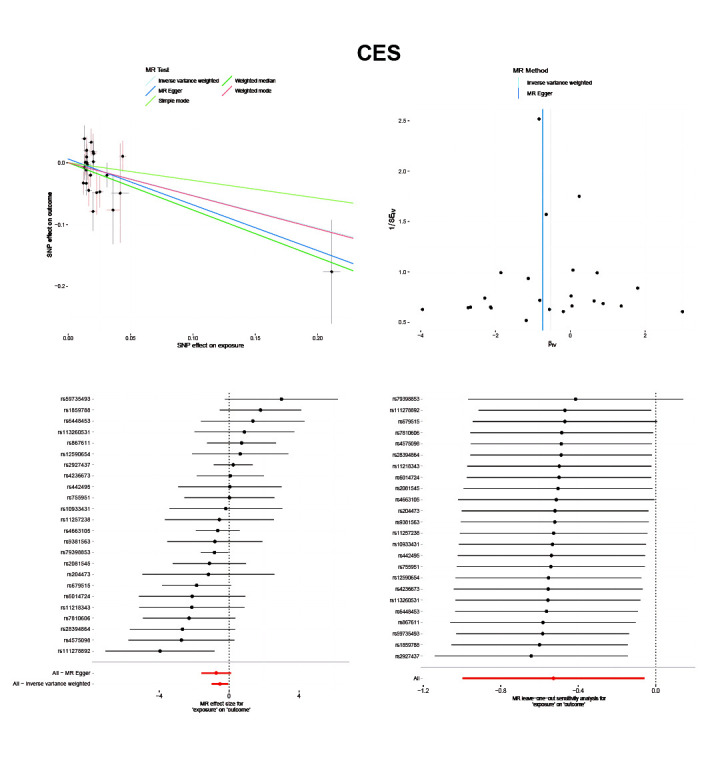


**Figure:**
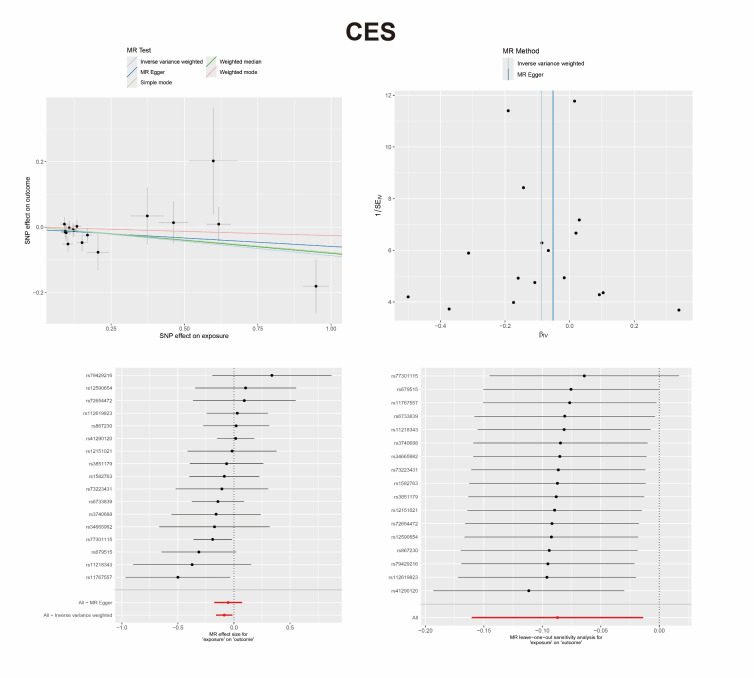


**Figure:**
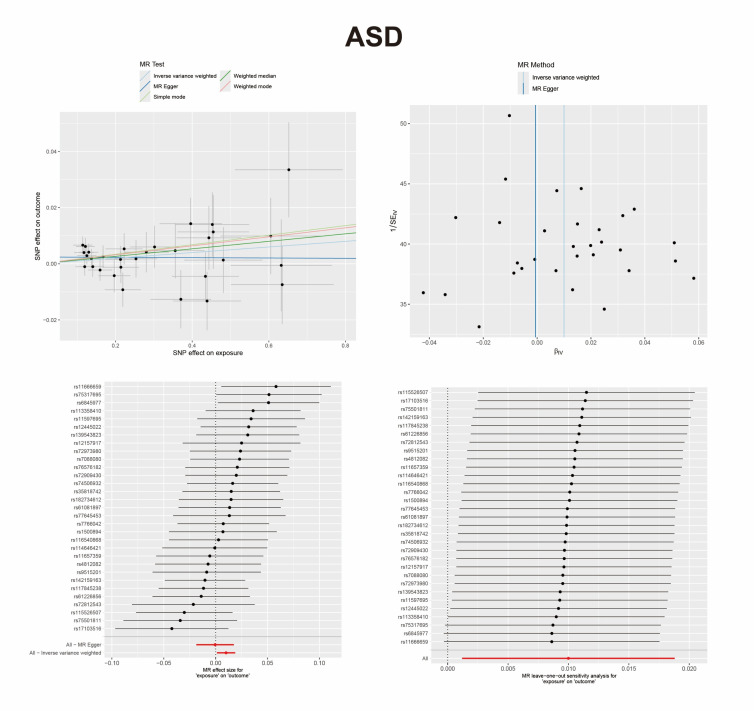


**Figure:**
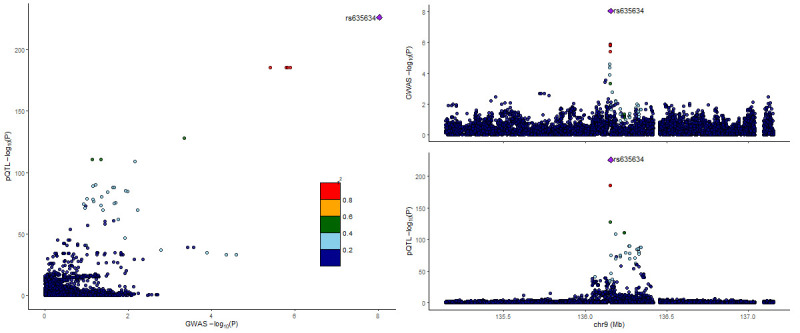


**Figure:**
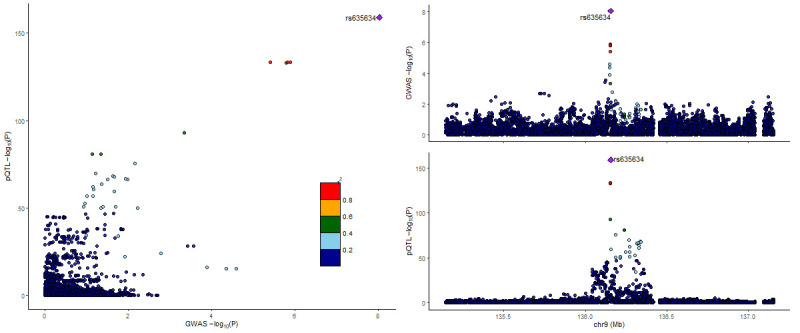


**Figure:**
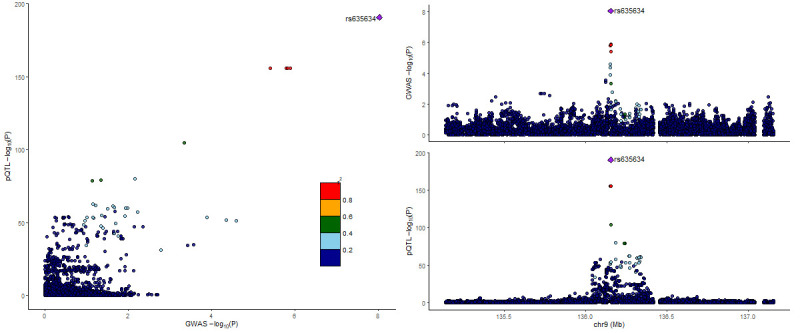


**Figure:**
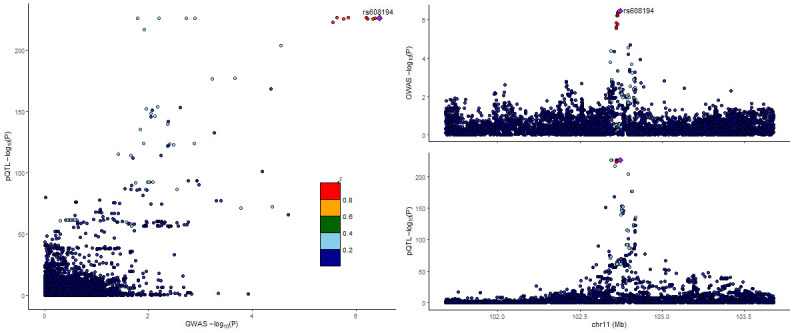


**Figure:**
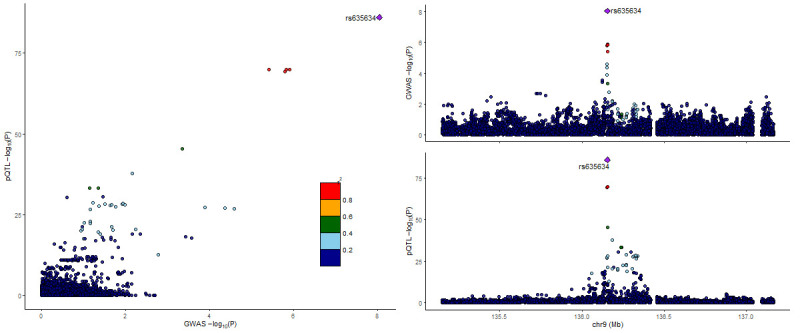


**Figure:**
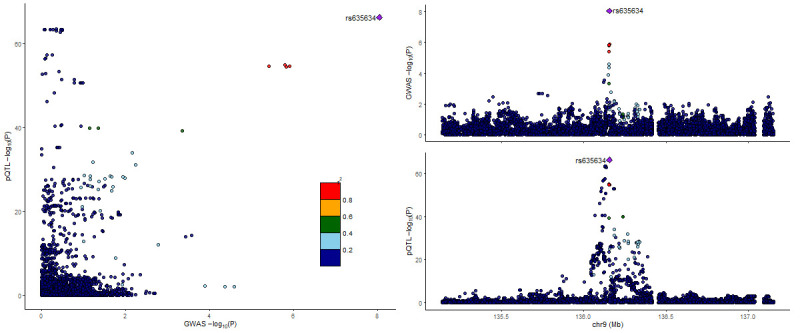


**Figure:**
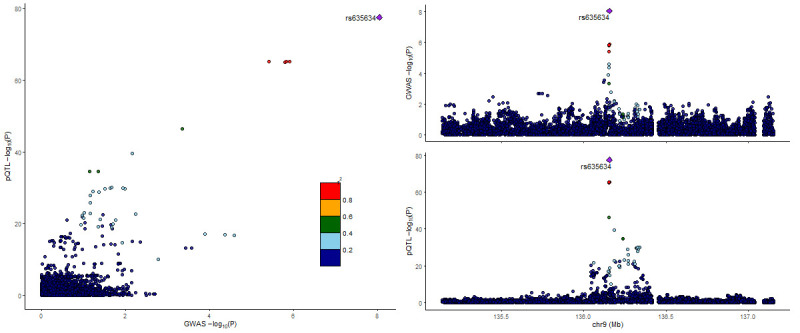


**Figure:**
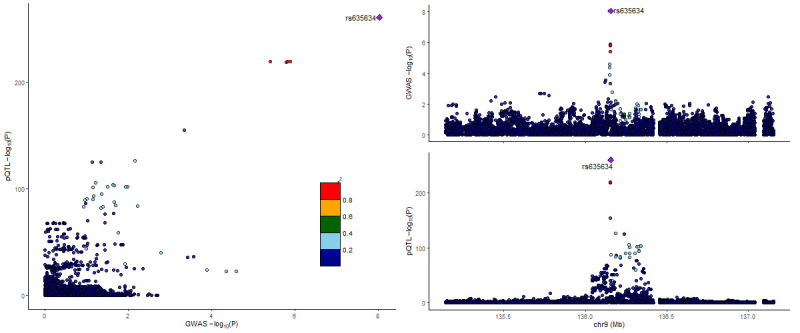


**Figure:**
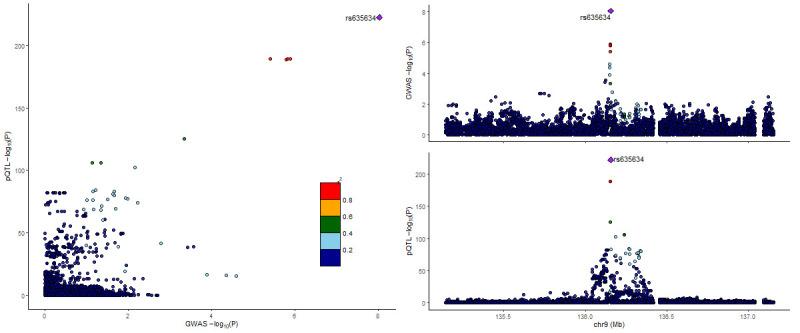


**Figure:**
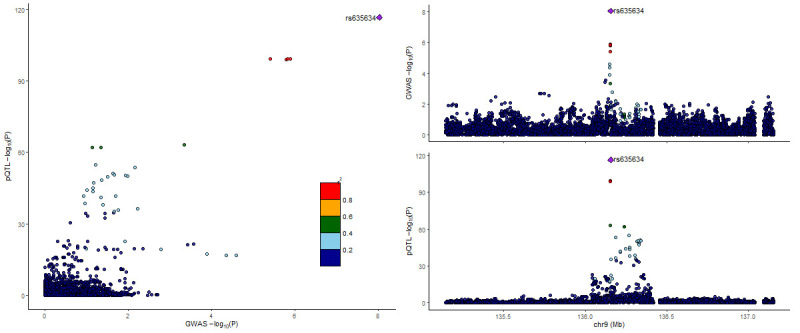


**Figure:**
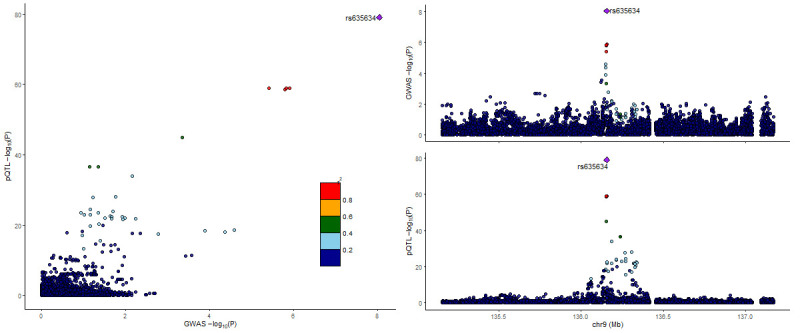


**Figure:**
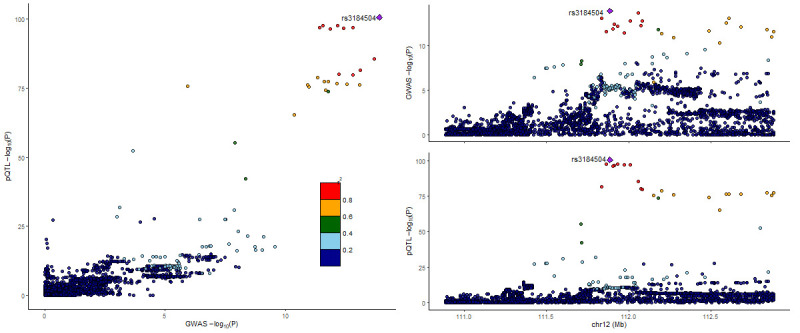


**Figure:**
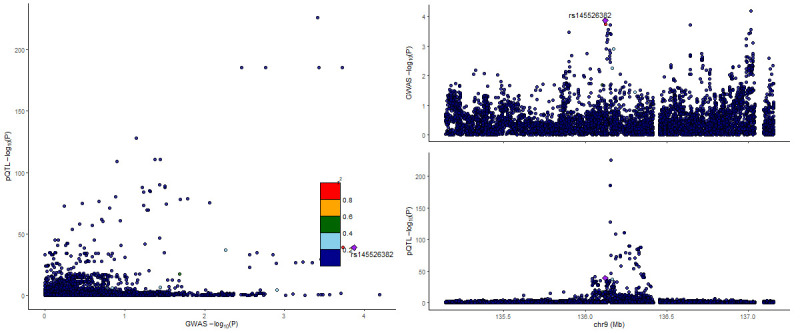


**Figure:**
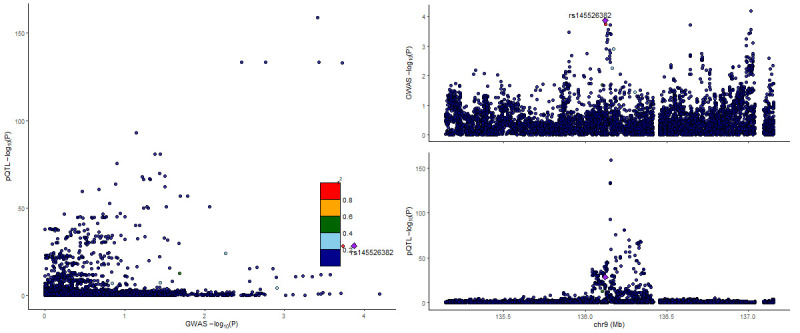


**Figure:**
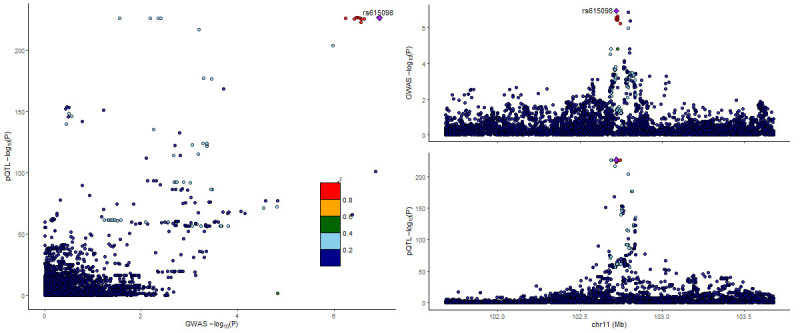


**Figure:**
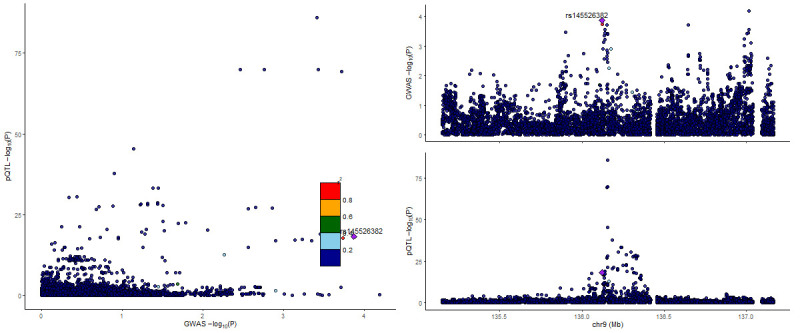


**Figure:**
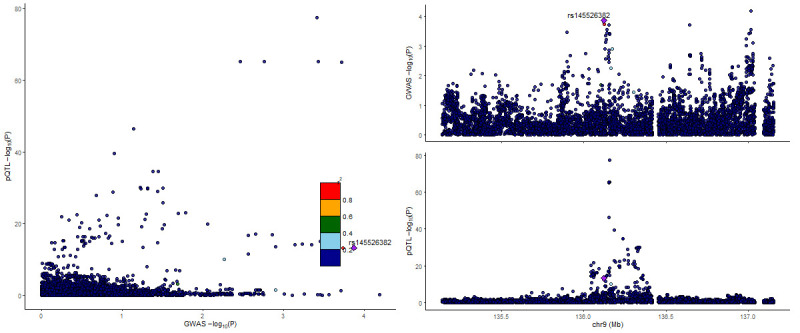


**Figure:**
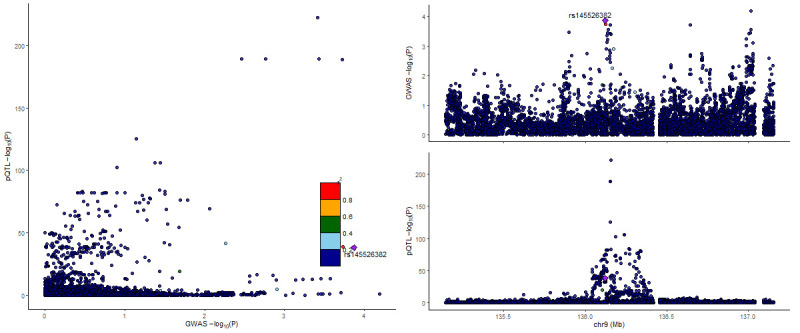


**Figure:**